# 5HT1AR-FGFR1 Heteroreceptor Complexes Differently Modulate GIRK Currents in the Dorsal Hippocampus and the Dorsal Raphe Serotonin Nucleus of Control Rats and of a Genetic Rat Model of Depression

**DOI:** 10.3390/ijms24087467

**Published:** 2023-04-18

**Authors:** Patrizia Ambrogini, Davide Lattanzi, Marica Pagliarini, Michael Di Palma, Stefano Sartini, Riccardo Cuppini, Kjell Fuxe, Dasiel Oscar Borroto-Escuela

**Affiliations:** 1 Department of Biomolecular Sciences, Università di Urbino Carlo Bo, I-61029 Urbino, Italy; davide.lattanzi@uniurb.it (D.L.); m.pagliarini1@campus.uniurb.it (M.P.); stefano.sartini@uniurb.it (S.S.); riccardo.cuppini@uniurb.it (R.C.); 2 Department of Experimental and Clinical Medicine, Faculty of Medicine and Surgery, Università Politecnica delle Marche, I-60121 Ancona, Italy; m.dipalma@staff.univpm.it; 3 Department of Neuroscience, Karolinska Institutet, 171 77 Stockholm, Sweden; kjell.fuxe@ki.se (K.F.); dasiel.borroto.escuela@ki.se (D.O.B.-E.); 4 Department of Human Physiology, Physical Education and Sport, Faculty of Medicine, University of Malaga, 29017 Malaga, Spain

**Keywords:** 5HT1A receptor, FGF receptor 1, heteroreceptor complexes, raphe, hippocampus, receptor-receptor interaction, GIRK channels, depression, neurophysiology

## Abstract

The midbrain raphe serotonin (5HT) neurons provide the main ascending serotonergic projection to the forebrain, including hippocampus, which has a role in the pathophysiology of depressive disorder. Serotonin 5HT1A receptor (R) activation at the soma-dendritic level of serotonergic raphe neurons and glutamatergic hippocampal pyramidal neurons leads to a decrease in neuronal firing by activation of G protein-coupled inwardly-rectifying potassium (GIRK) channels. In this raphe-hippocampal serotonin neuron system, the existence of 5HT1AR-FGFR1 heteroreceptor complexes has been proven, but the functional receptor–receptor interactions in the heterocomplexes have only been investigated in CA1 pyramidal neurons of control *Sprague Dawley* (SD) rats. In the current study, considering the impact of the receptor interplay in developing new antidepressant drugs, the effects of 5HT1AR-FGFR1 complex activation were investigated in hippocampal pyramidal neurons and in midbrain dorsal raphe serotonergic neurons of SD rats and of a genetic rat model of depression (the *Flinders Sensitive Line* (FSL) rats of SD origin) using an electrophysiological approach. The results showed that in the raphe-hippocampal 5HT system of SD rats, 5HT1AR-FGFR1 heteroreceptor activation by specific agonists reduced the ability of the 5HT1AR protomer to open the GIRK channels through the allosteric inhibitory interplay produced by the activation of the FGFR1 protomer, leading to increased neuronal firing. On the contrary, in FSL rats, FGFR1 agonist-induced inhibitory allosteric action at the 5HT1AR protomer was not able to induce this effect on GIRK channels, except in CA2 neurons where we demonstrated that the functional receptor–receptor interaction is needed for producing the effect on GIRK. In keeping with this evidence, hippocampal plasticity, evaluated as long-term potentiation induction ability in the CA1 field, was impaired by 5HT1AR activation both in SD and in FSL rats, which did not develop after combined 5HT1AR-FGFR1 heterocomplex activation in SD rats. It is therefore proposed that in the genetic FSL model of depression, there is a significant reduction in the allosteric inhibition exerted by the FGFR1 protomer on the 5HT1A protomer-mediated opening of the GIRK channels in the 5HT1AR-FGFR1 heterocomplex located in the raphe-hippocampal serotonin system. This may result in an enhanced inhibition of the dorsal raphe 5HT nerve cell and glutamatergic hippocampal CA1 pyramidal nerve cell firing, which we propose may have a role in depression.

## 1. Introduction

A wealth of brain functions, including mood, cognition, and emotions, are modulated by the serotonin (5HT) system [1,2], whose dysregulation has been implicated in the neuropathology of several psychiatric disorders, such as depression and anxiety [2]. The primary sources of 5HT in the brain are the raphe and para-raphe nuclei of the brainstem [3]. Indeed, serotonergic neurons, which send ascending projections to the telencephalon and diencephalon to release serotonin from high densities of 5HT nerve terminals to activate high-affinity synaptic and extra-synaptic receptors and channels via volume transmission [4,5], are located in these regions of the midbrain. The hippocampus is one of many brain regions that receives a dense serotonergic innervation originating from both median and dorsal raphe nuclei [6]; in this area, serotonin modulates hippocampal functions through the activation of distinct 5HT receptor subtypes expressed in excitatory and inhibitory neurons and in glial cells.

At least 15 major 5HT receptor subtypes [7,8] have been identified, and they are currently divided into seven classes (5HT1 to 5HT7) based on structural features and signaling mechanisms generally coupled to a G protein, except 5HT3 coupled to ion channels. Among these serotonin receptors, the 5HT1A receptors (R) have been extensively investigated for their role in the modulation of mood, anxiety, and cognition [9]. 5HT1AR are divided into two distinct classes based on their location: the 5HT1A auto-receptors, which are located on the soma and dendrites of serotonergic neurons in the midbrain raphe nuclei, and the 5HT1A post-junctional receptors located on non-5HT neurons, like the soma and dendrites of glutamatergic hippocampal pyramidal neurons and astrocytes [10]. 5HT1A auto-receptor activation induces a reduction in the firing rate of serotonergic neurons [11], thus decreasing activity-dependent serotonin release [12]. 5HT1A post-junctional receptor activation on pyramidal neurons also causes a decrease in neuronal firing activity [13]. It is documented that the inhibitory effect of 5HT1A receptors on the activity of both serotonergic nerve cells and hippocampal pyramidal neurons has to be ascribed to the activation of G protein-coupled inwardly-rectifying potassium (GIRK) channels that deeply hyperpolarize neurons [14,15] by an increase in K^+^ conductance [15,16].

It should be noted that 5HT1A receptors form a large number of heterocomplexes with other receptors in the plasma membrane [17]. Regarding this aspect, Borroto-Escuela and colleagues [18,19], using in situ Proximity Ligation Assay (in situ PLA), provided evidence for the existence of 5HT1AR-FGFR1 heteroreceptor complexes in the dorsal hippocampus and in the dorsal raphe and median raphe of the midbrain of *Sprague Dawley* (SD) control rats. In particular, this heteroreceptor complex has been shown to be involved in neuroplasticity in the rat hippocampus and in the midbrain raphe serotonergic neurons, mainly through the allosteric 5HT1A protomer enhancement of FGFR1 protomer signaling, leading to antidepressant-like actions [19]. In 2012, Borroto-Escuela and co-workers [19] found that acute and sustained intracerebroventricular (i.c.v.) infusion with FGF-2 and the 5HT1AR agonist 8-OH-DPAT in SD rats resulted in an improved antidepressant effect, evaluated using the forced swim test (FST), in comparison to 5HT1AR agonist treatment alone. It suggested a stronger and possibly more rapid antidepressant effect of the co-treatment than obtained with selective serotonin reuptake inhibitors (SSRIs), which specifically increase extracellular 5HT levels and are extensively used to pharmacologically treat mood disorders [20].

The proposed mechanism underlying the antidepressant-like actions obtained by co-activation of the two protomers forming the 5HT1AR-FGFR1 heterocomplex involves an uncoupling of the 5HT1AR Gi/o mediated opening of the GIRK channels. It is likely due to antagonistic allosteric interaction in the 5HT1AR-FGFR1 heterocomplex through which the agonist-activated FGFR1 protomer induces a conformational change in the 5HT1AR protomer, reducing its ability to open GIRK channels. In line with this evidence, our previous study [21] demonstrated that in SD rats, the activation of FGFR1 using a specific agonist is able to substantially reduce the opening of GIRK channels induced by the 5HT1A receptor in pyramidal neurons of the CA1 region of the dorsal hippocampus. Thus, the allosteric interaction between the 5HT1A receptors and FGFR1 protomers would prevent the hyperpolarizing effect of 5HT1A receptor activation on glutamatergic pyramidal neurons. This could explain possibly more rapid antidepressant effects of the co-activation of the two protomers in animal models of depression. However, the i.c.v. combined treatment over a 48 h period with FGF2 and 8-OH-DPAT in a genetic rat model of depression, *Flinders Sensitive Line* (FSL) rats of SD origin failed to produce any antidepressant effects in FST, suggesting that the synergistic effect of the co-treatment was missing in FSL rats [21].

Based on these findings and considering the impact of the allosteric receptor protomer interplay in possibly developing new, more rapidly acting antidepressant drugs targeting these heteroreceptor complexes, the current work aimed to gain an improved insight into the possible operation of 5HT1AR-FGFR1 heterocomplexes in the raphe-hippocampal serotonin system under control condition and in a genetic rat model of depression. To this purpose, the 5HT1AR-FGFR1 heterocomplex was studied in pyramidal neurons of the dorsal hippocampus, where it acts at post-synaptic/junctional level, and in the dorsal raphe serotonergic neurons of the midbrain involving the 5HT1Aauto-receptor-FGFR1 complexes, of SD and FSL rats.

## 2. Results

### 2.1. Electrophysiological Analysis of 5HT1AR-Activated GIRK Currents and Their Modulation by FGFR1 Agonist

#### 2.1.1. Hippocampal CA1 and CA2 Neurons in SD and FSL Rats

**Figure 1:**
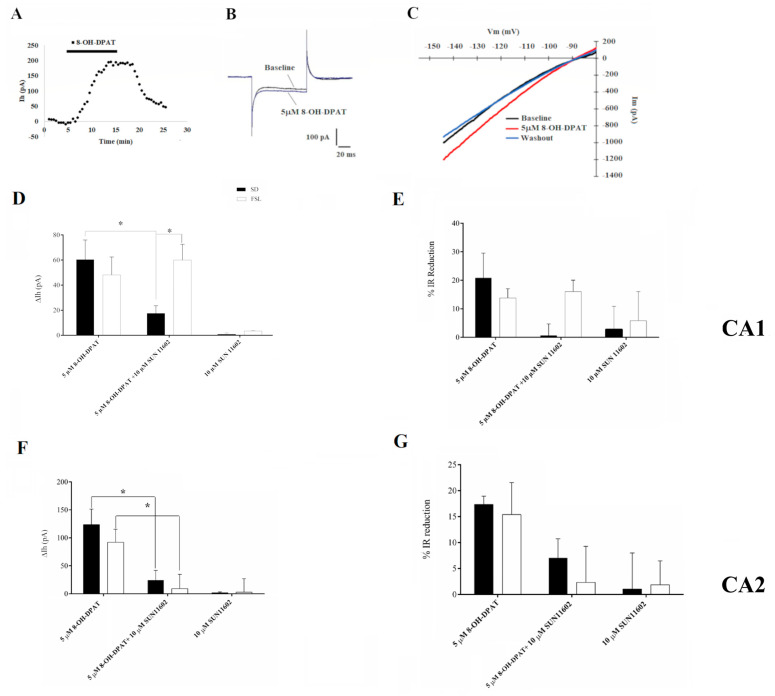
8-OH-DPAT-activated inwardly rectifying K^+^ conductance in CA1 and CA2 neurons of *Sprague Dawley* (SD) and *Flinders Sensitive Line* (FSL) rats. (**A**) Time-course of a representative experiment showing the effect on holding current (Ih) at −70 mV of 5HT1A receptor agonist 8-OH-DPAT bath application mediated by inwardly rectifying K^+^ conductance in CA2 hippocampal neurons. (**B**) Representative trace showing that 8-OH-DPAT application induced an input resistance (IR) decrease, suggesting a membrane channel opening in CA2 hippocampal neurons. (**C**) Representative current-voltage (Im-Vm) correlation plot. Traces are recorded before (Baseline; black line), during agonist application (5 µM 8-OH-DPAT; red line), and after washout (Washout; blue line). (**D**) Summary graph including agonists tested on CA1 neurons. Combined application of 5 µM 8 OH-DPAT together with FGFR1 agonist 10 µM SUN 11602 significantly reduced the amplitude of the GIRK current, measured at −120 mV, induced by 5HT1AR activation in SD CA1 pyramidal neurons, but not in FSL CA1 pyramidal neurons. Two-way analysis of variance (ANOVA) F (2, 44) = 8.473, *p* = 0.0008; Tukey’s post hoc: * *p* < 0.05. Number of recorded cells (n) SD: 8-OH-DPAT (8), 8-OH-DPAT + SUN 11602 (10), SUN 11602 (4); FSL: 8-OH-DPAT (12), 8-OH-DPAT + SUN 11602 (9), SUN 11602 (7). (**E**) GIRK channel opening decreased IR in SD CA1 neurons. As expected, while combined agonist treatment tended to reduce the IR drop elicited by 5HT1AR induced GIRK activation in SD rats, in FSL rats, there were no changes in IR. Number of recorded cells (n) SD: 8-OH-DPAT (8), 8-OH-DPAT + SUN 11602 (10), SUN 11602 (4); FSL: 8-OH-DPAT (12), 8-OH-DPAT + SUN 11602 (9), SUN 11602 (7). (**F**) Combined application of 5 µM 8 OH-DPAT together with FGFR1 agonist 10 µM SUN 11602 in CA2 hippocampal neurons significantly reduced the amplitude of the GIRK current induced by 5HT1AR activation in SD and FSL neurons. Two-way analysis of variance (ANOVA) F (2, 43) = 10.12, *p* = 0.0002; Tukey’s post hoc: * *p* < 0.05. (**G**) GIRK channel opening decreased input resistance in CA2 neurons. As expected, combined agonist treatment tended to reduce the IR drop elicited by 5HT1AR-induced GIRK activation both in SD rats and in FSL rats. Two-way analysis of variance (ANOVA) F (2, 44) = 3.686, *p* = 0.0331. Number of recorded cells (n) SD: 8-OH-DPAT (8), 8-OH-DPAT + SUN 11602 (6), SUN 11602 (5); FSL: 8-OH-DPAT (10), 8-OH-DPAT + SUN 11602 (10), SUN 11602 (10). All data are expressed as mean ± SEM.

GIRK channels represent the main effectors of 5HT1A receptors in different types of neurons, such as serotonergic raphe neurons and pyramidal CA1 neurons [15,21]. Based on our previous findings [21], we verified the existence of a 5HT1AR-activated GIRK-mediated current in CA1 and CA2 pyramidal neurons and its modulation by 5HT1AR-FGFR1 heteroreceptor complex activation in our experimental groups. Voltage-clamp experiments performed in SD rats showed an outward GIRK current induced by a ten-minute-long bath application of 5 µM 8-OH-DPAT both in CA1 neurons, confirming our previous results [21], and CA2 nerve cells, which partially reversed upon agonist washout (Figure 1A). The 8-OH-DPAT-induced outward GIRK current was associated with a decreased input resistance, indicating channel opening (Figure 1B). Very similar results were obtained by recording CA1 and CA2 neurons in FSL rats, even though the amplitude of GIRK channel-mediated current elicited by 5HT1A receptor activation tended to be smaller than that recorded in SD rats (Figure 1D,F). In both experimental groups, the 8-OH-DPAT-induced GIRK outward current matched the potassium reversal potential of –100 mV estimated considering the free potassium concentration inside (126 mM) the cell and outside (2.5 mM). The current–voltage relationship slope was steeper at hyperpolarized potentials in the voltage ramp protocol due to GIRK channel-induced inward rectifying response (Figure 1C). These results support the presence of Gi/o mediated GIRK current in CA1 and CA2 hippocampal neurons of SD and FSL rats.

FGFR1 activation in CA1 and CA2 neurons of SD and FSL rats, following the application of the specific agonist SUN 11602 (10 µM), did not affect the holding current (Figure 1D,F), pointing out its failure in GIRK channel opening. Co-application of 5 µM 8-OH-DPAT and 10 µM SUN 11602 to activate 5HT1AR-FGFR1 heteroreceptors significantly reduced 5HT1AR-induced GIRK currents (Figure 1D,F) and counteracted the IR decrease elicited by 5HT1AR-induced GIRK activation (Figure 1E,G) in CA1 and CA2 neurons of SD rats. Conversely, in CA1 neurons of FSL rats, the co-application of the agonists mentioned above induced a GIRK current similar to that obtained with 5 µM 8-OH-DPAT alone (Figure 1D), and, consistently, did not contrast the IR reduction caused by GIRK channel opening. On the other hand, CA2 neurons of FSL rats responded to the application of the agonist mixture in the same way as pyramidal neurons of SD rats (i.e., showing a reduced 5HT1AR-induced GIRK current (Figure 1F) and a more modest decrease of IR (Figure 1G). After washing the drugs out of the bath, all the observed effects were reversed.

**Figure 2:**
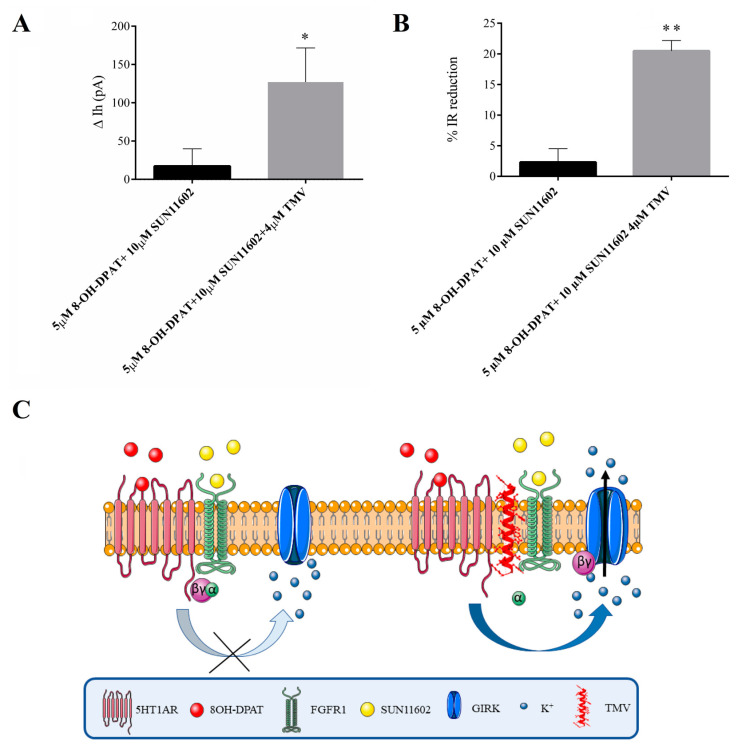
(**A**) TMV interfering peptide disrupts the receptor–receptor interaction within the 5HT1AR-FGFR1 heteroreceptor complex and significantly restores the hyperpolarizing current mediated by GIRK channel opening in CA2 of FSL rats. All data are expressed as mean ± SEM. Unpaired Student’s *t*-test * *p* < 0.05. Number of recorded cells (n): 8-OH-DPAT + SUN 11602 (8), SUN 11602 8-OH-DPAT + SUN 11602 + TMV (7). (**B**) TMV interfering peptide-mediated restoration of the GIRK channel opening mechanism reduces input resistance in CA2 neurons from FSL rats. All data are expressed as mean ± SEM. Unpaired student *t*-test ** *p* < 0.001. Number of recorded cells (n): 8-OH-DPAT + SUN 11602 (10), SUN 11602 8-OH-DPAT + SUN 11602 + TMV (7). (**C**) Cartoon showing the mechanism of action of the TMV interfering peptide: the peptide prevents the interaction between 5HT1A receptor and FGFR1 by overcoming the heteroreceptor-mediated inhibition of GIRK channel opening and favoring neuronal hyperpolarization.

In order to verify the evidence we found in the CA2 neurons of FSL rats, we applied the agonist mixture to CA2 neurons to activate the 5HT1AR-FGFR1 heteroreceptors in the presence of the TMV interfering peptide (a peptide representing V TM segment at residues 192–217 for human 5HT1A; UniProt identifying number: P08908; 4 µM) in the recording solution to prevent the interaction between 5HT1AR and FGFR1 [18] and, thus, the modulation of the GIRK-mediated hyperpolarizing currents. Before starting with the application of the TMV interfering peptide in the recording solution together with the mixture of the specific protomer agonists, we verified the specificity of this peptide on some hippocampal neurons by applying the peptide alone. Since we did not record any membrane current variation, we decided to proceed with the experiments concerning the application of TMV together with the mixture of the specific protomer agonists. Our findings showed that functional decoupling mediated by the interfering peptide significantly restored GIRK currents (Figure 2A), and increased the IR reduction (Figure 2B), demonstrating that these currents are modulated by the activity of the 5HT1AR-FGFR1 heteroreceptor complex (Figure 2C).

#### 2.1.2. Dorsal Raphe Serotonergic Neurons

**Figure 3:**
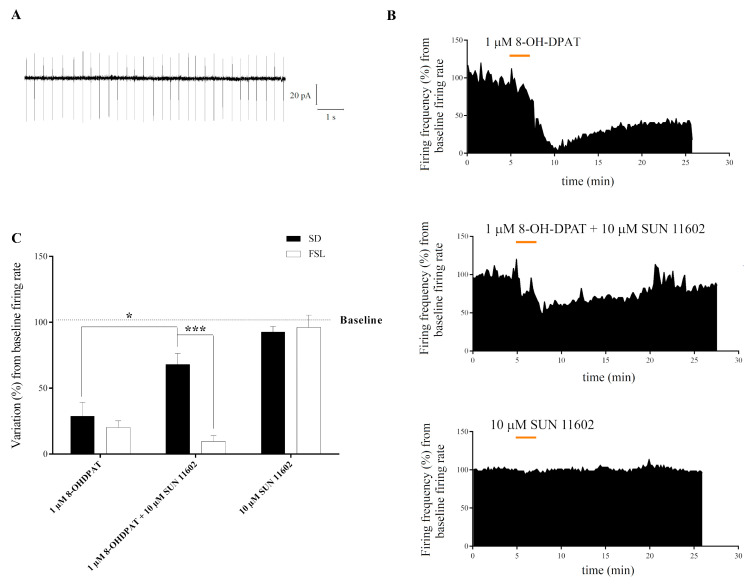
Analysis of dorsal raphe nucleus (DRN) neuron firing properties after 5HT1AR and/or FGFR1 agonist bath application. (**A**) Trace shows a segment of the loose-patch recording explaining the firing characteristics of the recorded DNR serotonergic neurons. (**B**) Representative time—course variation of DRN neuron firing activity after 2-min-long bath application of 1 µM 8-OH-DPAT, 10 µM SUN 11602, or a combination of them. (**C**) Variation of DRN neuron firing frequency induced by bath application of 1 µM 8-OH-DPAT and/or 10 µM SUN 11602 in *Sprague Dawley* (SD) and Flinders Sensitive Line (FSL) rats. All data are expressed as mean ± SEM. Two-way analysis of variance (ANOVA) F (2, 38) = 51.48, *p* < 0.0001; Tukey’s post hoc: * *p* < 0.05; *** *p* < 0.001. Number of recorded cells (n) SD: 8-OH-DPAT (6), 8-OH-DPAT + SUN 11602 (6), SUN 11602 (6); FSL: 8-OH-DPAT (11), 8-OH-DPAT + SUN 11602 (7), SUN 11602 (8).

5HT1A receptor activation in dorsal raphe neurons induces a hyperpolarizing current by activating GIRK channels [15]. This hyperpolarization can modify the serotonergic neuron autorhythmic firing activity, thus controlling the release of serotonin on target neurons. For this reason, the effects of 5HT1AR and FGFR1 agonists on the spontaneous firing activity of dorsal raphe serotonergic neurons were assessed in SD and FSL rats using the loose-patch approach. Neurons with a stable firing activity showing a frequency ranging from 2 to 4 Hz (Figure 3A) were considered. A two-minute-long bath application of 1 µM 8-OH-DPAT induced a drastic decrease in spontaneous firing in both groups of rats, which was maintained for at least 20 min and then partially reversed after the end of the application (Figure 3B). No difference was found between SD and FSL rats. On the contrary, in SD rats only, a co-application of 1 µM 8-OH-DPAT and 10 µM SUN 11602 counteracted the neuronal firing decrease (Figure 3B,C). However, the FGFR1 activation by the application of the specific agonist SUN 116052 (10 µM) did not affect spontaneous neuron firing in both animal groups (Figure 3B,C). These data led us to hypothesize that 5HT1AR-induced GIRK current in dorsal raphe serotonergic neurons could be modulated by the co-application of 5HT1AR and FGFR1 agonists in SD rats and that this modulation does not occur in FSL serotonergic neurons.

**Figure 4:**
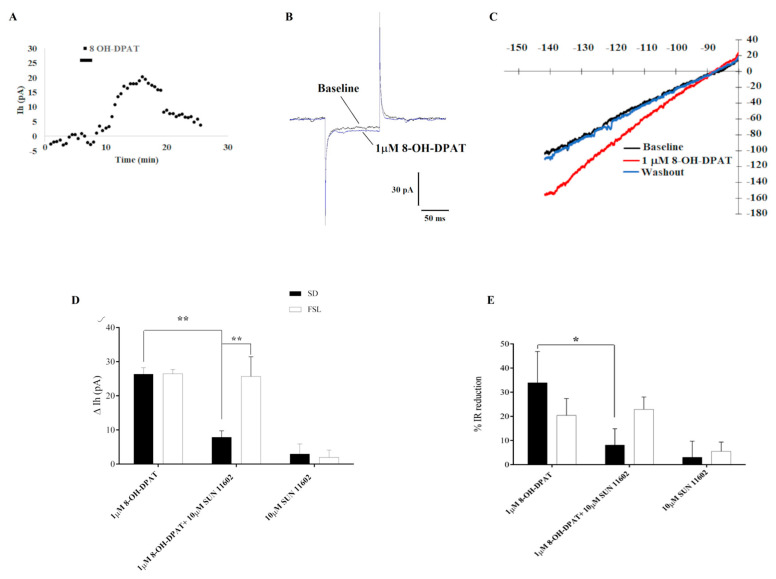
8-OH-DPAT-activated inwardly rectifying K^+^ conductance in dorsal raphe nucleus (DRN) serotonergic neurons. (**A**) Time-course of a representative experiment showing the effect on holding current (Ih) at −70 mV of 5HT1A receptor agonist 8-OH-DPAT bath application mediated by inwardly rectifying K^+^ conductance. (**B**) 8-OH-DPAT application induces an input resistance (IR) decrease, suggesting a membrane channel opening. (**C**) Representative current-voltage (Im-Vm) correlation plot. Traces are recorded before (Baseline; black line), during agonist application (1 µM 8-OH-DPAT; red line), and after washout (Washout; blue line). (**D**) Whole-cell patch-clamp analysis of DNR neurons showed that the activation of 5HT1AR induced an outward Gi/o-mediated current in DRN serotonergic neurons due to GIRK channel opening. Two-minutes-long combined application of 1 µM 8-OH-DPAT together with 10 µM SUN 11602 significantly reduced the amplitude of the 5HT1AR-induced GIRK current at −120 mV in SD but not in FSL rats. Two-way analysis of variance (ANOVA) F (2, 31) = 26.82, *p* < 0.0001; Tukey’s post hoc: ** *p* < 0.01. Number of recorded cells (n) SD: 8-OH-DPAT (5), 8-OH-DPAT + SUN 11602 (7), SUN 11602 (4); FSL: 8-OH-DPAT (7), 8-OH-DPAT + SUN 11602 (7), SUN 11602 (7); (**E**) GIRK channel opening decreased IR of DRN neurons. Combined agonist treatment tended to reduce the IR drop elicited by 5HT1AR-induced GIRK activation in SD rats but not in FSL. Two-way analysis of variance (ANOVA) F (2, 32) = 5.213, *p* = 0.0110; Tukey’s post hoc: * *p* < 0.05. Number of recorded cells (n) SD: 8-OH-DPAT (5), 8-OH-DPAT + SUN 11602 (7), SUN 11602 (4); FSL: 8-OH-DPAT (6), 8-OH-DPAT + SUN 11602 (8), SUN 11602 (8). All data are expressed as Mean ± SEM.

To verify this hypothesis, using the whole-cell patch-clamp technique, we measured the GIRK current and its modulation by the aforementioned agonists in dorsal raphe serotonergic neurons of SD and FSL rats. In our experimental conditions, a two-minute-long bath application of 1 µM 8-OH-DPAT elicited a small outward current at V-holding of −70 mV that reversed upon the agonist washout (Figure 4A) in both groups of rats. As described for CA1 and CA2 neurons, input resistance tended to also decrease in serotonergic neurons during 8-OH-DPAT application, indicating GIRK channel opening (Figure 4B). Furthermore, voltage ramp protocols showed a potassium-like reversal potential and an inward rectifier nature of the 8-OH-DPAT-induced current (Figure 4C). A bath application of the specific FGFR1 agonist, SUN 11602 (10 µM), did not affect the holding current in SD and FSL rats, suggesting that FGFR1 activation could not open ion channels. The combined application of 1 µM 8-OH-DPAT and 10 µM SUN 11602 resulted in a significantly lower holding current when compared to the 1 µM 8-OH-DPAT application alone (Figure 4D) in SD rats, indicating that 5HT1AR-FGFR1 heterocomplex activation was able to reduce GIRK current amplitude in dorsal raphe serotonergic neurons in this group of rats as it occurs in CA1 and CA2 hippocampal neurons. This mechanism possibly underlies the effect exerted by agonist co-application on the spontaneous firing activity of serotonergic neurons in SD rats. On the contrary, in FSL serotonergic neurons, the agonist co-application did not reduce the activity of the 5HT1A receptor on the GIRK channels, suggesting a disturbance in the 5HT1AR-FGFR1 heteroreceptor complex operation. This result is consistent with the lack of an effect that heterocomplex activation has in counteracting the 5HT1AR-induced serotonergic neuron firing activity decrease. The measurement of membrane input resistance supported the difference found in the GIRK current elicited by agonist co-application between SD and FSL rats (Figure 4E).

#### 2.1.3. Synaptic Plasticity Evaluation

**Figure 5:**
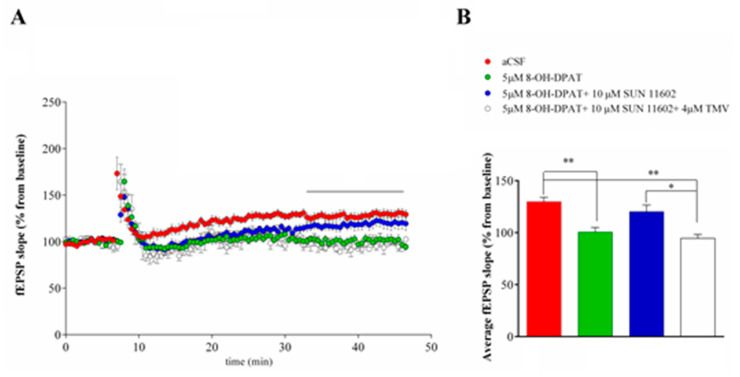
(**A**) A field Excitatory PostSynaptic Potential (fEPSP) slope (10% and 80% of max) recorded before and after the Schaffer Collateral high-frequency stimulation in SD rats. Values were normalized to the mean value obtained over the last 20 min of the baseline period and expressed as a percentage of this baseline value (n = 9 slices for aCSF, n = 7 for 5 µM 8-OH-DPAT, n = 10 slices for 5 µM 8-OH-DPAT + 10 µM SUN 11602, n = 4 for 5 µM 8-OH-DPAT + 10 µM SUN 11602 + 4 µM TMV). Two-way ANOVA test: F (3, 26) = 10.04, *p* < 0.01. Tukey’s multiple comparisons test: aCSF vs. 5 µM 8-OH-DPAT, *p* < 0.05; aCSF vs. 8-OH-DPAT + 10 µM SUN 11602 + 4 µM TMV, *p* < 0.05; 5 µM 8-OH-DPAT vs. 5 µM 8-OH-DPAT + 10 µM SUN 11602, *p* < 0.05; 5 µM 8-OH-DPAT + 10 µM SUN 11602 vs. 8-OH-DPAT + 10 µM SUN 11602 + 4 µM TMV, *p* < 0.05 from 26 to 40 min (black line) of recording post HFS. (**B**) Summary of mean ± SEM of fEPSP slopes from 35 to 40 min post-HFS. One-way ANOVA test: F (3, 26) = 7.999, *p* < 0.01. Tukey’s multiple comparisons test: * *p* < 0.05, ** *p* < 0.01.

**Figure 6:**
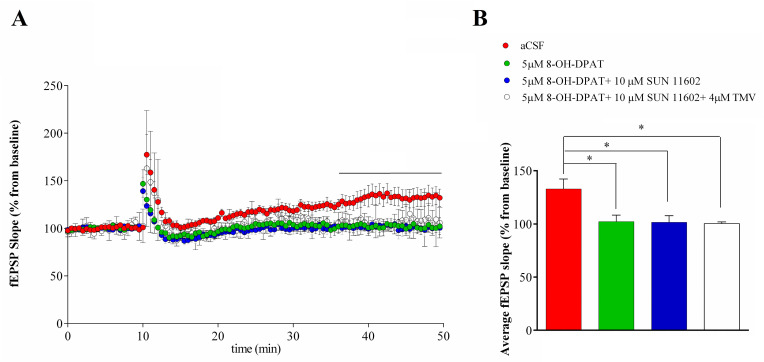
(**A**) A field Excitatory PostSynaptic Potential (fEPSP) slope (10% and 80% of max) recorded before and after the Schaffer Collateral high-frequency stimulation in FSL rats. Values were normalized to the mean value obtained over the last 20 min of the baseline period and expressed as a percentage of this baseline value (n = 6 slices for aCSF, n = 7 for 5 µM 8-OH-DPAT, n = 7 slices for 5 µM 8-OH-DPAT + 10 µM SUN 11602, n = 4 slices for 5 µM 8-OH-DPAT + 10 µM SUN 11602 + 4µM TMV). Two-way ANOVA test F (3, 21) = 4.2585, *p* < 0.05. Tukey’s multiple comparisons test: aCSF vs. 5 µM 8-OH-DPAT, *p* < 0.05; aCSF vs. 8-OH-DPAT + 10 µM SUN 11602, *p* < 0.05; aCSF vs. 5 µM 8-OH-DPAT + 10 µM SUN 11602 + 4 µM TMV, *p* < 0.05 from 30 to 40 min (black line) of recording post HFS. (**B**) Summary of mean ± SEM of fEPSP slopes from 35 to 40 min post-HFS. One-way ANOVA test: F (3, 21) = 4.980, *p* < 0.01. Tukey’s multiple comparisons test: * *p* < 0.05.

A high-frequency stimulation (HFS) of the stratum radiatum induced a comparable LTP in SD (Figure 5) and FSL rats (Figure 6) that reached a stable value about 30 min after HFS. In SD rats, LTP induction was fully prevented by 8-OH-DPAT application; on the contrary, the co-application of 5HT1AR and FGFR1 agonists did not block LTP induction (Figure 5), but when the receptor agonists were applied when adding the TMV interfering peptide to the mixture, LTP did not develop (Figure 5). In FSL rats, a bath application of 8-OH-DPAT alone or co-application of 8-OH-DPAT and SUN 1602 with or without the TMV interfering peptide induced the complete inhibition of LTP (Figure 6).

As expected, the activation of 5HT1A receptors induced LTP inhibition both in SD and FSL rats, likely due to GIRK channel-mediated hyperpolarization of CA1 neurons. Consistently with whole-cell experiment results in SD rats, 5HT1AR-FGFR1 co-activation allowed LTP induction to not elicit GIRK currents. Differently, in FSL rats the co-application of agonists made LTP induction by eliciting GIRK currents impossible.

## 3. Discussion

The existence of direct interactions between plasma membrane receptors, namely allosteric receptor–receptor interactions, has been assessed through several different methodologies, spanning from classical biochemical approaches to biophysical techniques [22,23]. Allosteric receptor–receptor interactions in the heteroreceptor complexes can give diversity, specificity, and bias to the receptor protomers due to the allosterically induced conformational changes in discrete domains leading to alterations in receptor protomer function and pharmacology. Consequently, allosteric receptor–receptor interactions in receptor complexes have attracted much attention within medicine for their promising potential as novel targets for treatment of neurological and mental disorders. In particular, even if the involvement of the G protein-coupled receptors (GPCRs) in these diseases has been known for a long-time, recent compelling evidence of GPCR heteroreceptor complexes have provided a novel understanding of the molecular mechanisms involved [24,25]. The causal link between maladaptive synaptic signaling and a misbalanced or even dysfunctional expression of heteroreceptor complexes has been extensively reported in many diseases, including depression [17]. In this regard, the 5HT1AR-FGFR1 heteroreceptor complex may play a relevant role in the pathogenetic mechanisms of major depressive disorder [18,19,26].

The presence of these heterocomplexes has been ascertained by using different types of techniques, such as in situ PLA, co-immunoprecipitation, and BRET, in cellular models and in pyramidal neurons of rat dorsal hippocampus [18], and their presence was ascertained later on in midbrain serotonergic dorsal and median raphe neurons [19]. It is well documented that 5HT1A receptor activation induces GIRK channel opening, causing plasma membrane hyperpolarization, due to a K^+^ conductance increase [15,27,28]. Based on this evidence, from a functional point of view, it is likely that the activation of the FGFR1 protomer through allosteric receptor–receptor interactions can modulate the ability of 5HT1A receptor protomer to affect the GIRK channel-mediated current. Therefore, using GIRK current as an electrophysiological readout, it is possible to study FGFR1 agonist-produced alterations of 5HT1AR agonist-induced modulation of GIRK currents and consequent input resistance variation. In keeping with this view, we found [21] that the outcome of the 5HT1AR-FGFR1 interaction resulted in the reduction of the 5HT1AR hyperpolarizing action due to GIRK channel opening, possibly inducing an increased pyramidal neuron firing in the CA1 field of rat hippocampus.

In this scenario, the current work, using the same electrophysiological approach, extends our previous findings, providing, for the first time, evidence for: (i) a functional interaction between 5HT1AR and FGFR1 protomers in plasma membrane heteroreceptor complexes of CA2 pyramidal neurons, besides those of CA1, and dorsal raphe serotonergic neurons in control rats; (ii) lack of functioning of the heterocomplex in CA1 pyramidal neurons, and dorsal raphe serotonergic neurons from a genetic model of depression; (iii) the preservation of the heterocomplex functioning in CA2 neurons of depressed rats. Since 5-HT1AR-FGFR1 heterocomplexes are located at the somatodendritic level, they ideally mediate serotonin effects on neuronal firing, both as auto and as post-junctional heteroreceptor complexes.

In DRN neurons, the 5HT1AR-FGFR1 heterocomplexes act as auto-receptors, and, when activated, they can reduce the inhibition mediated by the 5HT1AR activation on the firing activity of serotonergic neurons, allowing the level of serotonin that reaches the different projection areas to raise. By using a combination of 5HT1AR and FGFR1 receptor agonists, we observed the following modulation in DRN neurons of SD rats, compared to the sole activation of 5HT1AR: (i) the minor decrease in neuronal firing frequency; (ii) the lower whole-cell recorded GIRK current; and (iii) the minor reduction in membrane resistance. Altogether, these findings are consistent with the existence of the 5HT1AR-FGFR1 heteroreceptor in DRN neurons of control rats and provide evidence of its functionality. Conversely, failure of the same receptor agonist mixture to alter the 5HT1A receptor capacity to activate GIRK channels observed in the genetic model of depression, FSL rats, indicates the heteroreceptor ineffectiveness due to its absence in raphe nuclei or the disruption of receptor–receptor interaction. In this view, a hyperfunctioning of GIRK channel in FSL rats, due to the lack of a functioning 5HT1AR-FGFR1 heteromer able to modulate potassium current, might be the basis for the depressed-like behavior of FSL rats. In line with this suggestion, blockers of the GIRK channels are considered to be antidepressants [29,30].

In keeping with this evidence, the differences found between SD and FSL rats might be explained by the theory that depression may develop as a result of the 5HT1AR-FGFR1 heterocomplex being inactive or dysfunctional. These changes may be linked to the onset of depression because they may result in lower levels of serotonin reaching the raphe’s projection regions. Based on the monoaminergic theory of depression, there would be a deficiency in the serotonergic system’s operation, as well as the emergence of the classic signs of the depressive syndrome [31]. Moreover, our unpublished results show that DRN serotonergic neurons of FSL rats have excitability characteristics different from those of the controls, which lead these neurons to have a low firing frequency that could be associated with a consequent lower release of serotonin on specific targets. According to the literature, depressed animals exhibit lower serotonin levels [32], which may be the cause of cellular adaptation events that would lead to lower numbers of pre- and post-synaptic heteroreceptors [33]. Consistently with the hypothesis that the existence of the functioning heteroreceptor serves to avoid excessive hyperpolarization of pre- and postsynaptic neurons, the uncoupling of the two protomers in FSL rats might be related to less serotonin production and, therefore, a reduced requirement to minimize hyperpolarization. In addition, previous studies have shown that FGF2 can act as an anxiolytic and anti-depressive agent in rodents and that decreased levels of hippocampal FGF2 and FGF2 receptors have been found in postmortem brains of individuals with mood disorders [34]. Thus, the existence of sufficient levels of serotonin and FGF2 might be considered a prerequisite for the development of the receptor heteromerization as well.

Serotonergic neurons of DRN reach pyramidal neurons of the hippocampus making up the raphe-hippocampal pathway [3], which is thought to have a role in the pathophysiology of depressive disorder. In this scenario, the modulation of GIRK currents by 5HT1AR-FGFR1 heteromer activation may result in an antidepressant effect. In hippocampus pyramidal neurons, the 5HT1AR-FGFR1 heterocomplexes act as post-junctional receptors, thus influencing the effects of serotonin on hippocampal neuronal firing. In the current study, we found that FGFR1 activation decreases GIRK-mediated currents brought on by the administration of a 5HT1A receptor agonist in the CA1 and CA2 pyramidal neurons of control rats. The 5HT1AR protomer capacity to activate GIRK channels may be reduced in hippocampal neurons, as in DRN nerve cells, because of the FGFR1 agonist binding to the receptor, which is thought to reduce hyperpolarization and increase neuronal activity. On the other side, the analysis performed on the CA1 neurons of FSL rats reveals a different situation, where the co-application of the 5HT1AR and FGFR1 specific agonists does not determine a reduction of the GIRK hyperpolarizing current, indicating a defect in the heteroreceptor’s functioning in the depressed phenotype. This is further supported by data from the analysis of membrane input resistance in the CA1 of FSL rats. These findings are consistent with behavioral studies done on FSL rats, which do not appear to benefit from the antidepressant effect owing to activation of the 5HT1AR-FGFR1 heteromer generated by intracerebroventricular injection of the combination of receptor protomer agonists, as occurs in controls [21].

It is worth considering the particular case of CA2 pyramidal neurons, which behave equally both in SD and in FSL rats. Indeed, FGFR1 activation reduces in the same way GIRK-mediated currents produced by 5HT1A receptor agonist in CA2 neurons of the control and depressed rats. The use of a peptide representing V TM segment (residues 192–217) for human 5HT1AR (UniProt identifying number: P08908), known to prevent the interaction between FGFR1 and 5HT1AR in RN33B cell cultures, allowed us to take a look into the mechanism of interaction between the 5HT1A receptor and FGFR1, as well as to unveil the implication of the heteroreceptor in the modulation of currents mediated by GIRK channels in hippocampal pyramidal neurons of CA2 in FSL rats. Overall, these experiments provided evidence of the effective receptor–receptor interaction between the 5HT1AR and FGFR1 protomers within the heterocomplex when activated by specific receptor agonists, and in particular, they showed us the preservation of the receptor–receptor interaction in CA2 neurons of depressed rats. In the past, the CA2 region was simply defined as the area located between the CA3 and the CA1 regions, showing large pyramidal cells that, differently from CA3 neurons, lack complex spines on the apical dendrites and direct innervation from the dentate gyrus. In the last decade, growing evidence indicates that CA2 neurons have peculiar electrophysiological features [35,36,37,38,39] markedly different from CA1 and CA3 nerve cells, suggesting that they are engaged differently in their response to synaptic inputs. Very recently, Lopez-Rojas et al. [40] unveiled a cortical circuit conveying social information to CA2, indicating that CA2 neurons integrate information from other extrahippocampal circuits to locally compute social memory. In line with this evidence, it has been demonstrated that the CA2 region has its own set of connections, structures, and gene expression patterns, suggesting that it likely serves a unique function within the hippocampus [41]. Therefore, the different behavior of CA2 neurons we found out in the depressed animals compared to CA1 nerve cells could be imputable to the peculiar characteristics of this distinct hippocampal region.

While taking into account the function that CA2 plays in the regulation of social relationships, the 5HT1AR-FGFR1 heteromer results to retain its function in hippocampal CA2 neurons in depressed rats, suggesting a different implication of these neurons in the raphe-hippocampal pathway involved in depression. Therefore, the mechanism underlying the lack of an antidepressant effect of receptor agonist treatment seen in vivo in the genetic model of depression may involve a disruption in allosteric receptor–receptor interactions in the 5HT1AR-FGFR1 heteromer in hippocampal neurons of CA1. Because of an altered 5HT1AR-FGFR1 heteroreceptor activity in FSL rats, the CA1 pyramidal neurons would become susceptible to stronger GIRK currents in the presence of serotonin, which causes a greater hyperpolarization of the neurons themselves. Due to this, even if neurons are adequately stimulated, they could be unable to generate post-synaptic excitatory potentials necessary to trigger the LTP process. The capacity to transfer information from short-term memory into long-term memory is one of the major functions of the LTP event in the hippocampus, as well as to retrieve information stored in long-term memory [42]. Important preclinical evidence suggests that depression may disrupt the normal balance between LTP and LTD in pyramidal neurons, favoring the latter, particularly in the murine hippocampus [43,44,45,46].

The 5HT1AR and FGFR1 coactivation promotes their heterodimerization, enhancing the recruitment of β-arrestins to the heterocomplex and boosting the internalization of the complex [47]. It is well recognized that β-arrestins are essential for a number of cellular processes, such as facilitating signal transduction pathways and desensitizing membrane receptors. Another feature of 5HT receptor signaling is suggested by the finding that receptor endocytosis is required for 5HT1A receptor-mediated activation of the ERK cascade [48]. The ERK pathway is the MAPK signaling pathway that has received the most attention in research about depression. In post-mortem investigations, it was discovered that MDD patients had reduced Raf-ERK1/2 signaling in the PFC and hippocampus, as well as decreased hippocampal MEK5-ERK5 signaling [49,50]. Ras-dependent signals can be transmitted by the same processes that prevent the 5HT1A receptor from binding to G proteins. In addition to their involvement in the desensitization of GPCRs, β-arrestins have been shown to function as scaffolding elements that modulate receptor signaling. As a result, β-arrestins can connect GPCRs and signaling proteins without the necessity for G protein-mediated activation [51]. In light of this, the inability to trigger LTP in depressed rats in presence of the receptor agonist mixture could be due to the convergence of effects caused by heteroreceptor malfunction in the CA1 pyramidal neurons: lower neuronal activity, due to increased activation of the GIRK channels, and, on the other hand, decreased internalization of the complex mediated by β-arrestin recruitment, resulting in reduced activation of the pERK 1/2 pathway, which is required for synaptic plasticity processes. As a consequence, memory and learning deficits may result from this mechanism modifications in depressed patients.

Altogether, these functional investigations performed on the hippocampus and midbrain raphe indicate that synergistic allosteric receptor–receptor interaction develops within the 5HT1AR-FGFR1 heteroreceptor complex during coactivation by agonists. The formation of these complexes is hypothesized to contribute to the antidepressant effects by recruiting 5HT1A receptors and triggering their dissociation from GIRK channels. As a result, the neurons could be more depolarized due to the reduction of 5HT1A auto-receptor activity in the midbrain 5HT neurons, as well as improved neurotransmitter release in other areas of the brain, such as the hippocampal area. The modification in the heteroreceptor activity seen in FSL rats may be a contributing factor to depression or may be the result of adaptive mechanisms put in place in the brain as a response to a depressive disease.

To fully understand this scenario and validate the assumptions generated by the findings of the present work, further investigations on the possible conformational changes that the 5HT1AR and FGFR1 undergo in raphe neurons and CA1 pyramidal neurons of depressed rats are needed. Although extrapolation from rats to humans is always difficult and fraught with errors, present findings suggest a possible involvement of 5HT1AR-FGFR1 heteroreceptor complexes in the depressive disorders.

## 4. Materials and Methods

### 4.1. Animals

Experiments were performed using 2–3-month-old male SD and FSL rats. They were reared and group-housed in a temperature (21 ± 1 °C) and humidity (50 ± 5%) controlled vivarium room with a 12 h/12 h light/dark cycle (7:00 A.M. to 7:00 P.M. lights on). Food and water were available ad libitum. Before experiments, FSL rats were tested in forced swim test [21] to verify the depressive behavior. The use of animals was in compliance with the current Italian legislation (D.lgs 26/2014) on animal experimentation, which is in strict accordance with the European Council Directives on animal use in research (n. 2010/63/EU) (protocol codes: BEF09.N.4CO and BEF09.N.O0C, approved by the Institutional Review Board of Italian Health Ministry). All efforts were made to minimize animal suffering and to reduce the number of animals used.

### 4.2. Electrophysiology Proceedings

#### 4.2.1. Slice Preparation

For electrophysiological experiments, rats (SD: n = 26 from different litters; FSL: n = 32 from different litters;) were anesthetized with isoflurane and sacrificed by decapitation; brains were quickly removed and incubated in chilled oxygenated solution containing in mM: 110.0 choline Cl^−^, 2.5 KCl, 1.3 NaH_2_PO_4_, 25.0 NaHCO_3_, 0.5 CaCl_2_, 7.0 MgCl_2_, 20.0 dextrose, 1.3 Na^+^ ascorbate, 0.6 Na^+^ pyruvate, 5.5 kynurenic acid (pH: 7.4; 320 mOsm). Hippocampal transversal slices (400 μm thick) or brainstem coronal slices (300 μm thick) were obtained by vibrating microtome (Campden Instruments, Loughborough, UK) and allowed to recover in oxygenated artificial Cerebrospinal Fluid (aCSF) containing in millimolar: 125.0 NaCl, 2.5 KCl, 1.3 NaH_2_PO_4_, 25.0 NaHCO_3_, 2.0 CaCl_2_, 1.3 MgCl_2_, 1.3 Na^+^ ascorbate, 0.6 Na^+^ pyruvate, 10.0 dextrose (pH: 7.4; 320 mOsm).

The slices were kept in steadily oxygenated aCSF for at least 1h at room temperature (experiments in CA2) or 3 h (experiments in dorsal raphe nucleus—DRN) to allow cell recovery before electrophysiological recordings. Slices were then transferred into a recording chamber where they were continuously superfused throughout the electrophysiological recordings with oxygenated aCSF at a rate of 3 mL/min [52,53].

#### 4.2.2. CA1 and CA2 Pyramidal Neuron Whole-Cell Analysis

Compelling evidence has demonstrated that serotonin 5HT1A receptors are able to open GIRK channels, generating a hyperpolarizing outward potassium current [27,28]. Thus, 5HT1A receptor activation and its modulation related to 5HT1AR-FGFR1 heteroreceptor complex formation can be analyzed using whole-cell patch-clamp technique, as previously described [21]. In brief, whole-cell electrophysiological recordings were performed under visual guidance using a Zeiss (Carl Zeiss Oberkochen, Germany) Axioskop microscope equipped with an infrared camera connected to a monitor and an Axopatch-200B amplifier and WinWCP V5.4.0 software for data acquisition and analyses. The recording pipettes were filled with an internal solution containing in mM: 126 potassium gluconate, 8 NaCl, 0.2 EGTA, 10 HEPES, 3 Mg2ATP, 0.3 GTP (pH = 7.2; 290 mosM). Correction was made for junction potential between internal and external solutions.

Somata of neurons were identified in the CA1 and CA2 pyramidal cell layer based on their typical shape and localization. After the whole-cell configuration was established, we measured, in voltage-clamp mode, input resistance (IR) and membrane capacitance (C) by recording holding current (Ih) in response to 300 ms, 5 mV hyperpolarizing step. Regarding CA2 neurons, the high membrane capacitance and low resistance recorded (229.8 ± 28.6 pF; 242.8 ± 27.5 MΩ, respectively) were also used to confirm their identity, according to the literature [39]. The Ih variation was recorded in voltage-clamp mode, holding the membrane potential at −70 mV.

We quantified the opening of GIRK channels, evaluating the occurrence of: (i) an IR decrease; (ii) a positive increase in the Ih, necessary to maintain the membrane potential at −70 mV; (iii) and an inward current increase at hyperpolarized potentials (about −110/120 mV) by applying hyperpolarizing voltage ramps. GIRK current dynamics were analysed using single or combined treatment with the agonists for the two receptors under investigation to assess whether 5HT1AR-FGFR1 interaction might result in a modulation of 5HT1AR activity. In particular, after breaking into whole-cell configuration, once Ih reached a stable value, the 5HT1AR agonist 8-OH-DPAT (5 μM; BioTechne s.r.l., Milan, Italy) and/or selective FGFR1 agonist SUN 11602 (10 μM; BioTechne s.r.l., Italy) was applied in the perfusion bath. Finally, in a set of experiments, a peptide representing V transmembrane (TM) segment (residues 192–217) for human 5HT1AR (UniProt identifying number: P08908), known to prevent the interaction between FGFR1 and 5HT1A in RN33B cell cultures, was added (TMV, 4 µM) in the perfusion bath.

#### 4.2.3. Loose-Seal Cell-Attached Recordings in Dorsal Raphe Nucleus (DRN) Serotonergic Neurons

Neurons within DRN were visualized using a Zeiss Axioskop microscope equipped with an infrared camera connected to a monitor. Recordings were carried out using an Axopatch-200B amplifier and WinWCP V5.4.0 software for data acquisition and analyses. Neurons selected for recordings were localized based on the rat brain atlas of Paxinos and Watson and identified by their typical firing properties [54]. Electrodes were pulled from thick-walled borosilicate and filled with aCSF. After positioning the pipette in contact with the cell membrane, development of loose seal was monitored by recording the firing activity of the cell. When it was possible to record a spontaneous firing activity, gentle suction was slowly applied until detected spikes increased their amplitude on the background noise, and neurons showing a firing frequency out of the considered range (2–4 Hz) were discarded. The firing frequency was calculated by measuring the number of spikes in 10-s intervals. After the recording of a 5 min long steady baseline, we performed a single or combined bath application of the 5HT1AR agonist 8-OH-DPAT (1 μM) and/or selective FGFR1 agonist SUN 11602 (10 μM) for 2 min. We decided to use 1 µM 8-OH-DPAT to avoid complete firing inhibition and to allow the recovering of autonomous activity during the wash-out phase of serotonergic dorsal raphe neurons.

#### 4.2.4. DRN Serotonergic Neuron Whole-Cell Analysis

DRN neuron whole-cell recordings were carried out based on the protocol described above for hippocampal neuron analysis. Indeed, after the recording of a 5 min baseline using cell-attached patch-clamp mode to identify the serotonergic neurons showing 2–4 Hz firing frequency, we moved to the whole-cell recording configuration. Thus, when we obtained the recording of a baseline with a stable Ih, we applied to the recording bath the 5HT1AR agonist (1 μM 8-OH-DPAT), FGFR1 agonist (10 μM SUN 11602), or a mixture of them for 2 min to study GIRK current dynamics.

#### 4.2.5. Field Potential Recordings

Synaptic plasticity was investigated by evaluating the ability to elicit long-term potentiation (LTP) in Schaffer collateral-CA1 pathway of CTRL and FSL rats, as previously described [53]. Briefly, recording micropipettes and bipolar stimulating electrodes were placed in the stratum radiatum of CA1 with approximately 300 µm distance between them. Slices that produced field excitatory postsynaptic potentials (fEPSPs) of 1 mV or higher in amplitude were considered for experiments, and the stimulation intensity that evoked a half-maximal response was chosen for test pulse and tetanic stimulation. Low-frequency test pulses (at 30-s intervals) were applied to elicit baseline responses. Once obtained a stable baseline of approximately 20 min, the 5HT1AR agonist 8-OH-DPAT (5 μM) and/or selective FGFR1 agonist SUN 11602 (10 μM) were applied in the perfusion bath for 10 min. In a set of experiments, the TMV interfering peptide (4 µM) was also added to the receptor agonist mixture in the perfusion bath. After the wash-out, the medial perforant pathway was simulated using the LTP protocol consisting of a stimulus pattern of 3 trains of 100 Hz applied for 1 s separated by an interval of 20 s [55]. The fEPSP was then monitored by recordings for 40 min. Slope (between 10% and 80% of max) of the fEPSP was analyzed and taken as a measure of synaptic strength; values were normalized to the mean value obtained over the last 20 min of the baseline period and expressed as a percentage of this baseline value.

### 4.3. Data Analysis

Data are expressed as mean ± SEM. The number of samples (n) in each experimental condition is reported in figure captions. Data were analysed using the commercial program GraphPad PRISM 6.0 (GraphPad Software, San Diego, CA, USA). Statistical analysis was performed by one-way or two-way analysis of variance (ANOVA) followed by Tukey’s post hoc test. The significance threshold was established at *p* = 0.05.

## Data Availability

The data that support the findings of this study are available from the corresponding author upon reasonable request.
